# When a Forgotten Kidney Stone Breaks Free: An Unexpected Case of Spontaneous Nephrocutaneous Fistula

**DOI:** 10.7759/cureus.95735

**Published:** 2025-10-30

**Authors:** Usman I Shukr, Fatima Badar, Adee Hussain, Musab U Khalid

**Affiliations:** 1 Department of Urology, Fauji Foundation Hospital, Rawalpindi, PAK; 2 Department of Urology, Fatima Hospital, Wah Cantt, PAK

**Keywords:** calculus, fistula, nephrocutaneous, renal stone, renocutaneous, spontaneous expulsion

## Abstract

Nephrocutaneous fistula (NCF) is an exceptionally rare urological entity, most often arising as a late complication of neglected renal calculi, chronic pyelonephritis, or renal tuberculosis. Spontaneous extrusion of renal stones through such fistulous tracts is even less frequently documented. We describe the case of an 85-year-old woman who presented with spontaneous extrusion of a large staghorn calculus through a lumbar cutaneous opening. The patient had a decade-long history of untreated renal stones and a non-functioning kidney, repeatedly declining definitive surgical management. Imaging confirmed a fistulous tract connecting the kidney to the skin with residual calculi. Given her advanced age and comorbidities, she opted for conservative management with antibiotics, analgesia, and wound care. This case underscores the severe consequences of untreated renal calculi, highlights the diagnostic role of imaging in defining fistulous anatomy, and emphasizes the importance of timely surgical intervention, especially in cases of non-functioning kidneys, to prevent such rare but debilitating outcomes.

## Introduction

Renal calculi are a relatively common disease, with data suggesting that they occur in around 1 in 11 people at some time in their life [1]. Stone presentations are varied, and they may remain asymptomatic for a long time. Struvite stones, also known as staghorn calculi, are commonly associated with urease-producing organisms like Proteus species; they can lead to chronic inflammation, parenchymal destruction, and eventual fistula formation [2,3]. Spontaneous nephrocutaneous fistulae (NCFs) are very rare clinical manifestations involving an abnormal communication between the renal parenchyma and skin surface. Their occurrence has decreased significantly in developed countries due to early diagnosis, advanced surgical interventions, and improved infection control. Major etiologies include xanthogranulomatous pyelonephritis, renal tuberculosis, and long-standing obstructive uropathy due to renal calculi [2-5]. The spontaneous expulsion of a renal calculus through the fistula is an exceedingly rare occurrence. Herein, we present a unique case of a spontaneous nephrocutaneous fistula culminating in the expulsion of a sizable struvite calculus.

## Case presentation

**Figure 1:**
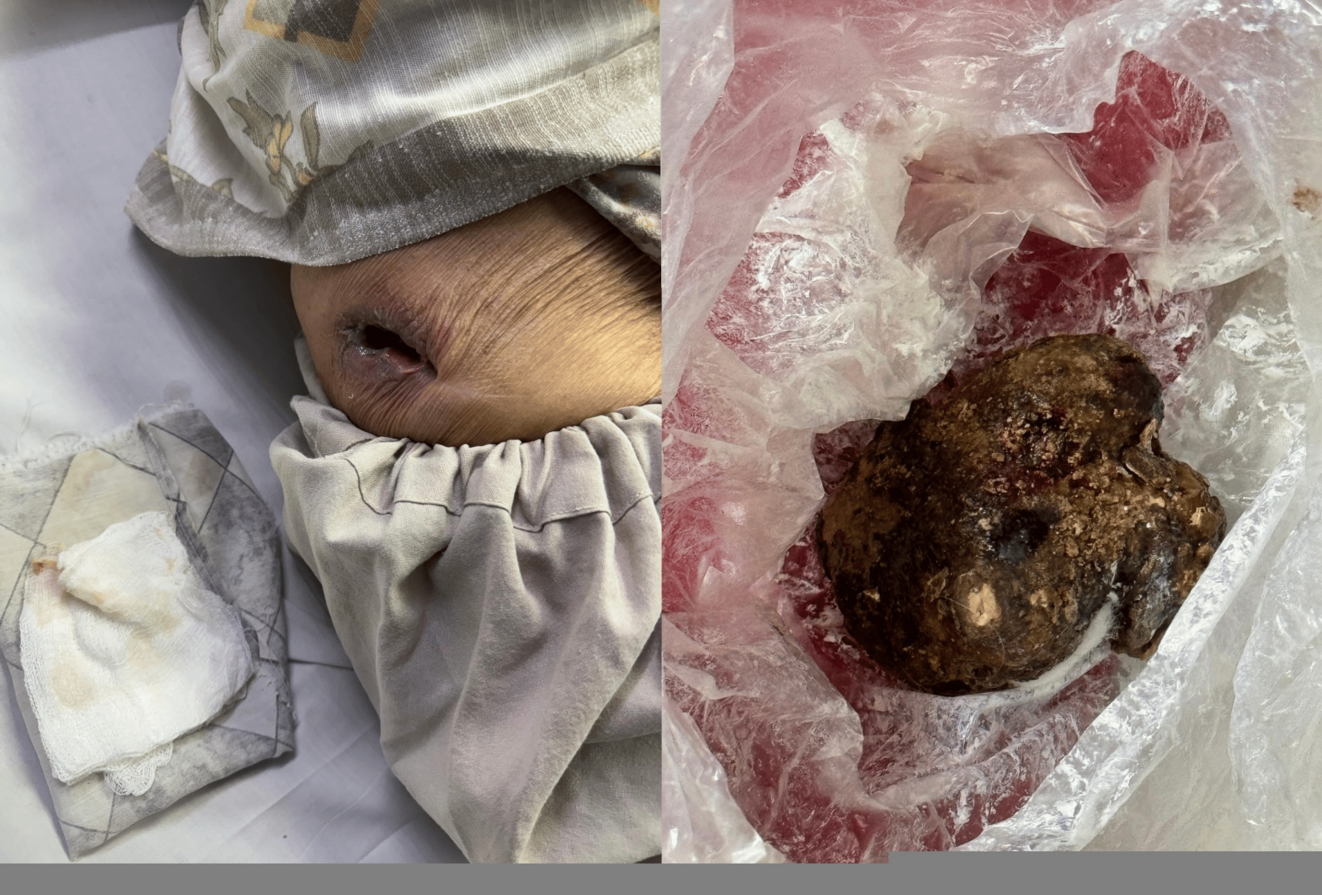
The wound at the time of presentation (left) and the expelled calculus (right).

An 85-year-old lady presented to the emergency department of Fauji Foundation Hospital, Rawalpindi, in December 2024 with severe colicky right flank pain and a spontaneous cutaneous opening in the right lumbar region, through which a large calculus had recently been expelled (Figure 1).

**Figure 2:**
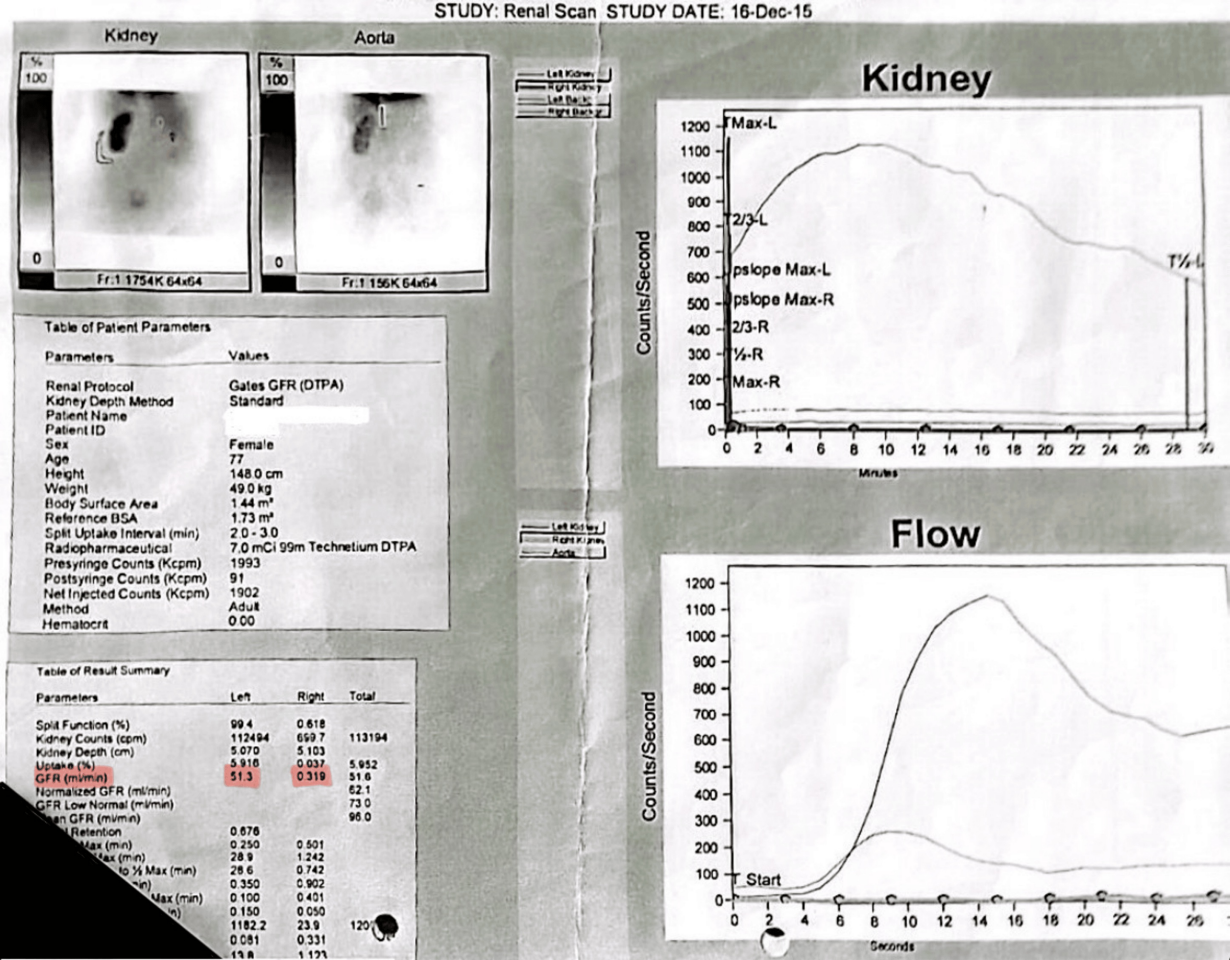
Diethylenetriaminepentaacetic acid (DTPA) scan of the patient performed in 2015. 99mTc-DTPA renal scintigraphy, which shows differential renal function. The graphs represent the tracer uptake and flow through the two kidneys, with the right kidney forming a flat curve representing poor renal function. The table in the lower left provides the exact functional data for each kidney, and GFR values are highlighted in red.

Her prior medical history included intermittent right-sided flank pain diagnosed in 2015. Imaging at that time revealed a non-functioning right kidney with multiple staghorn calculi, parenchymal thinning, pyonephrosis, and fluid tracking into the posterior abdominal wall. Diethylenetriamine pentaacetate (DTPA) scan confirmed a glomerular filtration rate (GFR) of 0.3 mL/minute in the affected kidney, while the contralateral kidney had a GFR of 51.3 mL/minute (Figure 2). Despite initial antibiotic therapy and percutaneous nephrostomy, the patient declined nephrectomy and was lost to follow-up.

In 2018, she presented again and had developed a persistent draining sinus at the right lumbar region, intermittently discharging pus. Despite clinical suspicion of a nephrocutaneous fistula, the patient again refused surgical management. She remained symptomatic and was treated conservatively with analgesics and antibiotics.

In her current presentation (2024), she was admitted and subsequently remained hemodynamically stable. Laboratory investigations are presented in Table 1.

**Table 1:** Laboratory findings at presentation.

Laboratory investigation	Results	Normal range
Serum urea	4.7 mmol/L	3.3-8.3 mmol/L
Serum creatinine	69 µmol/L	60-120 µmol/L
eGFR	73.9 mL/minute	>90 mL/minute
Leukocyte count	9.5 × 10^9^/L	4-10 × 10^9^/L
Serum uric acid	310 µmol/L	140-360 µmol/L
Pus culture	*Escherichia coli*, sensitive to cefoperazone/sulbactam	
Urine culture	*Escherichia coli*, sensitive to cefoperazone/sulbactam	

**Figure 3:**
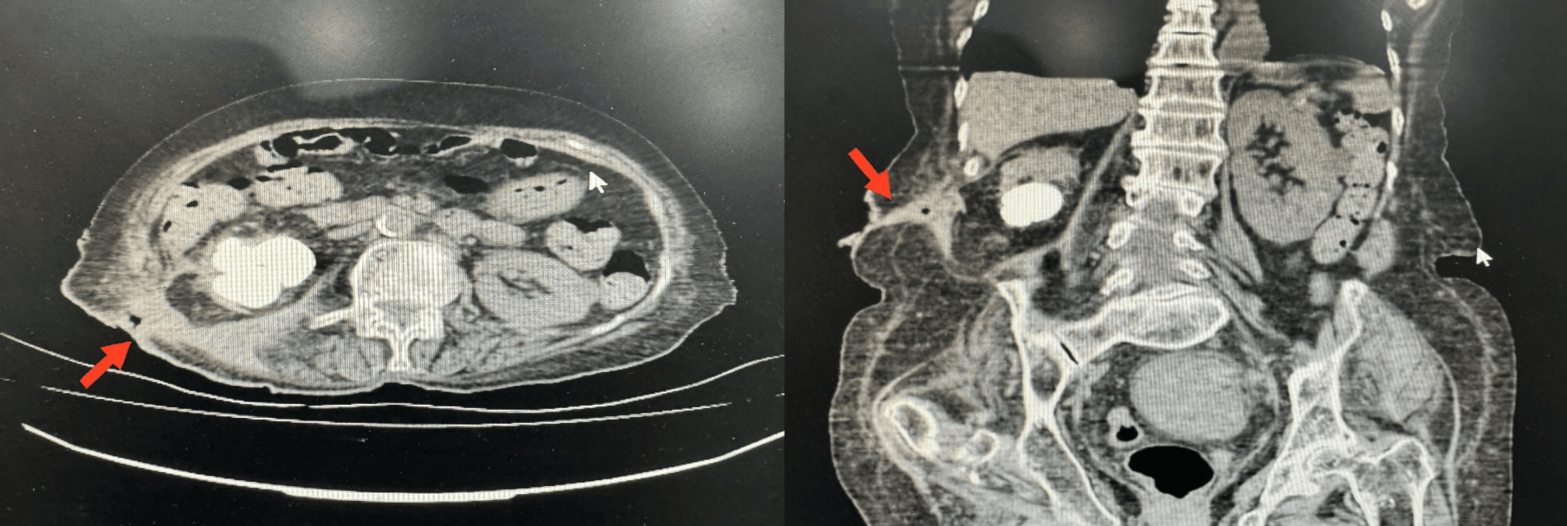
Axial (left) and coronal (right) CT scan sections at the level of the fistula. The axial section shows the extension of the fistulous tract from the right kidney to the skin. The coronal section gives a clearer view of the continuous fistula. The right kidney is shrunken with severe parenchymal thinning and multiple large calculi in the renal pelvis. Arrows indicate the location of the fistula in each image.

Renal ultrasonography revealed bilateral parenchymal changes and calculi. Non-contrast CT of the kidneys, ureters, and bladder (KUB) demonstrated a fistulous tract measuring 75 mm extending from the right perinephric space to the skin surface, with residual staghorn calculi and the largest stone measuring 4.5 × 4.3 × 3.2 cm (1357 HU) (Figure 3).

The combination antibiotic cefoperazone/sulbactam was initiated along with intravenous Acetaminophen for pain. Wound care involved regular irrigation and dressing. Given the patient’s age and operative risk, she again opted for conservative management. She was discharged in a stable condition.

## Discussion

Spontaneous extrusion of renal calculi through a fistula tract is exceptionally rare. Breatnach et al. first reported CT evidence of a staghorn calculus extruding into soft tissue [6]. A few other case reports since then have described the spontaneous passage of stones through a fistula. Two reports describe stone expulsion retroperitoneally into the psoas muscle [7,8]. Spontaneous passage of a stone outside the body has been documented even less. Chandrasekar et al. described a case of a staghorn calculus at the surface of a cutaneous fistula, which was manually extracted [2]. A case report from 2001 describes a male with spina bifida and complete extrusion of the calculus [9]. Our report describes a sizeable calculus being spontaneously expelled through a cutaneous fistula and, to the best of our knowledge, is the first case of its kind to be reported from Pakistan.

A nephrocutaneous fistula itself is an exceedingly rare occurrence. A review of the literature shows that common causes leading to its formation are chronic calculous pyelonephritis, xanthogranulomatous pyelonephritis, and renal tuberculosis [4,5,10,11]. Renal calculi are demonstrated in a majority of these cases; however, NCF formation is an uncommon presentation for renal calculous disease. Taking our patient and other reported cases into account, a common pattern involving neglected calculi, chronic infection, and non-functioning kidneys culminating in fistula formation can describe instances in which a fistula does form as a complication of calculous disease [2,3,11]. Pathophysiological mechanisms involve persistent inflammation, infection-induced tissue necrosis, epithelialization of sinus tracts, and sustained pressure gradients from obstructive stones, which together promote fistula formation. A positive culture for E. coli and a leukocyte count in the upper normal range point towards chronic infection and underlie the formation of a fistula in this case. Our case adds to the literature in describing the risk factors for NCF formation and highlighting a unique presentation of the disease. One can conclude that a nephrocutaneous fistula almost always forms on the background of a chronic disease process in the kidney, as a late complication after the kidney is rendered non-functioning. Thus, timely management of renal calculi can preclude their development. The finding of a non-functioning kidney and calculous disease should prompt swift management. 

For diagnosis, imaging, particularly CT, plays a pivotal role in defining the fistulous anatomy and guiding treatment planning. An algorithm developed by Rubilotta et al. also recommends this approach [12]. Non-contrast CT can easily be performed initially and provides visualization of the fistula. Contrast-enhanced CT can more accurately delineate the fistulous tract. Due to pre-existing renal insufficiency and to optimize stone visualization, non-contrast CT was preferred in our patient. Surgical nephrectomy and fistula excision remain the gold standard for definitive treatment in non-functioning kidneys and were the treatment of almost all patients when reviewing literature [4,8,10,13]. Conservative approaches such as drainage and antibiotics may suffice in high-risk surgical candidates when surgery is not possible, although long-term resolution is uncommon. Furthermore, conservative management carries risks like recurrent infection and sepsis. Nephron-sparing approaches like percutaneous glue embolization or flap-based surgical repair have been employed [14,15]. The renal function in these cases was preserved, and thus they may be considered in select patients. The management decision should always be made while keeping in mind the risks and benefits of each option in the particular patient being treated.

## Conclusions

This case exemplifies the rare but serious consequences of untreated or neglected renal calculi. Early identification and timely surgical management are essential to prevent complications like nephrocutaneous fistula. Clinicians in resource-constrained settings must maintain a high index of suspicion for such presentations. Definitive treatment through nephrectomy and fistula excision offers the best outcomes, but education and compliance remain integral to achieving successful resolution.
